# An *in vivo* confocal, prospective, masked, 36 months study on glaucoma patients medically treated with preservative-free or preserved monotherapy

**DOI:** 10.1038/s41598-019-41038-y

**Published:** 2019-03-12

**Authors:** Gemma Caterina Maria Rossi, Luigia Scudeller, Chiara Lumini, Alessandra Venera Mirabile, Erica Picasso, Federica Bettio, Gian Maria Pasinetti, Paolo Emilio Bianchi

**Affiliations:** 1 University Eye Clinic, Università degli Studi di Pavia, IRCCS Policlinico San Matteo Foundation, Pavia, Italy; 2 Clinical Epidemiology and Biometric Unit, Scientific Direction, Fondazione IRCCS Policlinico S. Matteo, Pavia, Italy; 3 Eye Unit, Istituto Beato Palazzolo, Bergamo, Italy

## Abstract

The aim of this study was to evaluate the *in vivo* effects at 3 years of preservative-free tafluprost on corneal health. It was a prospective, masked, study on consecutive patients with a new prescription of preservative-free (PF) tafluprost (naïve-N or switched-S, 44 and 14 patients), and preserved (P) bimatoprost 0.003% or travoprost 0.004% (P-group, 35 patients). A complete ophthalmic examination and an *in vivo* corneal confocal microscopy evaluation were performed at baseline and every 6 months for 3 years. Ninety-three patients were enrolled, clinical parameters were similar in the groups at baseline, apart from intraocular pressure (IOP) which was lower in the S-group (p = 0.012). Both at baseline and over time, confocal microscopy parameters had different trends. At baseline, keratocyte activation was similar in the three groups (p = 0.43) but over the next months naïve patients treated with PF-tafluprost presented a significant (p = 0.004) reduction in keratocyte activation. Sub-basal nerves tended to increase in patients switched to PF-tafluprost (p = 0.07) while were stable in the other two groups (p = 0.11 in PF and 0.40 in P group). Grade of tortuosity was stable over time in the three groups. Beading-like formations were stable over time for the P- and the PF-group, while significantly increased in the S-group (p = 0.027). Endothelial density values were statistically different at baseline (p = 0.007), they decreased both in PF-group and in S-group (p = 0.048 and 0.001, respectively), while increased in P-group (p = 0.006). Our study is the first to show that a PF-tafluprost formulation does not significantly alter the corneal structures as examined by confocal microscopy after 36 months of topical daily therapy, while improving corneal alterations due to chronic preserved therapies.

## Introduction

Glaucoma is a disease of the optic nerve: elevated intraocular pressure (IOP) has been recognized as the main risk factor for its development and progression. Several prospective randomized clinical studies have established the benefits of IOP reduction to prevent and control the damage of the optic nerve and the loss of vision (AGIS^1^, EMGT^2^, OHTS^3^, and EGPS^4^).

Most glaucomas are initially treated with topical agents to reduce aqueous humour production and/or to enhance the aqueous outflow.

As underlined by the European Glaucoma Society (EGS) guidelines^5^, the highest reduction of IOP is obtained with prostaglandins: this justifies the fact that most glaucoma patients are initially treated with a prostaglandin, as monotherapy. A meta-analysis has pointed out that differences among prostaglandins in the efficacy of reducing IOP did not exceed 1 mmHg while there are differences in tolerability^6^. Ocular side effects are common among patients treated with this class of drug and may exacerbate ocular surface disorders, caused by the active compound, the specific preservative, the excipients and the patient’s ocular surface status^7–10^. Considering that the EGS stated that “the goal of glaucoma treatment is to maintain the patient’s visual function and related quality of life at a sustainable cost (…) in terms of inconvenience and side effects”^5^, it should be kept in mind, when choosing a drug, to consider not only the efficacy and safety of a drug over the long term, but also patient’s characteristics.

Topical medical treatment often become chronic, and long term topical glaucoma medications may exacerbate or cause ocular surface disease: the correlation between the use of benzalkonium chloride (BAK) -preserved anti-glaucoma medications and signs/symptoms of ocular surface disease has been widely recognized, as well as the reduction of signs/symptoms after the switch to unpreserved drugs^9,11,12^. Since confocal microscopy allows clinical insights at the cellular level, providing quantitative data about the healthy and the inflammatory and toxic changes of the ocular surface, the instrument has been introduced in the clinical use to evaluate ocular surface status in glaucoma patients^13,14^.

To date most studies regarding ocular surface status evaluation in glaucoma patients refer to cross sectional studies while few studies have examined the effects of a specific therapy in a prospective way mainly for short periods^12,15,16^.

The primary aim of our study was to prospectively evaluate changes in corneal structures in the first 36 months of follow-up in glaucoma patients treated with preservative-free or preserved prostaglandins.

## Methods

### Study design

In this prospective cohort study, three groups of patients were enrolled over a period of 6 months: 1) newly diagnosed patients with primary open angle glaucoma (OAG) or normal tension glaucoma (NTG) needing a topical therapy to which preservative-free tafluprost 0.0015% (PF-group) has been prescribed; 2) newly diagnosed patients with open angle glaucoma (OAG) or normal tension glaucoma (NTG) needing a topical therapy to which preserved bimatoprost 0.003% or preserved travoprost 0.004% (P-group) have been prescribed; and 3) consecutive OAG or NTG on topical preserved prostaglandin or beta-blocker monotherapy needing a switch (S-group).

The study was carried out in accordance with the Declaration of Helsinki and after the approval of the Ethics Committee of the Fondazione IRCCS Policlinico San Matteo, Pavia, Italy (Proc. N. 20140031162). Subjects provided their signed informed consent before being enrolled. The study was not supported by any industry nor received any other form of financial grant or sponsorship.

### Eligibility criteria

Inclusion criteria were as follows: age 18 years or older, first diagnosis of early to moderate OAG or NTG (visual field MD <12 dB), no previous IOP-lowering topical therapy, for PF-group and P-group; age 18 years or older, previous diagnosis of early to moderate OAG or NTG (visual field MD <12 dB), previously preserved prostaglandin monotherapy needing a switch to a preservative free formulation (due to tolerability or efficacy reasons) for S-group.

Exclusion criteria were lack of consent; any ocular condition that was of safety concern or interfering with the study results; any ocular condition requiring the use of eye drops during follow-up (i.e., dry eye, uveitis); closed/barely open anterior chamber angles or history of acute angle closure; ocular surgery or argon laser trabeculoplasty within the preceeding 6 months; ocular inflammation/infection occurring within 3 months prior to pre-trial visit; hypersensitivity to BAK or to any other component of the trial drug solutions; any corneal pathology; diabetes at any stage; other abnormal condition or symptom preventing the patient from entering the trial (need for more than one IOP-lowering treatment), according to the investigator’s judgment; refractive surgery patients; women who were pregnant, of childbearing potential and not using adequate contraception or nursing; inability to adhere to treatment/visit plan.

### Diagnostic criteria

The diagnosis of OAG required IOP >21 and <32 mmHg on at least two visits; the presence of the glaucomatous optic nerve head (ONH), confirmed by an expert fundus examination; and at least two reliable Humphrey 24-2 full threshold visual field tests performed on different days.

The diagnosis of NTG required the presence of glaucomatous ONH, confirmed by an expert fundus examination; and at least two reliable Humphrey 24-2 full threshold visual field tests performed on different days with GHT outside normal limits, independently of the IOP value.

### Planned assessments

All subjects underwent a complete ophthalmic examination comprehensive of visual field and *in vivo* corneal confocal microscopy examination every 6 months for a period of 36 months.

The same glaucoma specialist evaluated the IOP (GCMR); and the same corneal specialist performed the confocal microscopy (FB); doctors involved in evaluating the patients were masked to patient’s therapy.

IOP (the mean of three consecutive measurements) was recorded with the Goldmann tonometer; all IOP values were recorded in the morning (between 9 a.m. and 12 noon) at each visit, with the last dose assumed to be in the previous evening at 9 p.m.

### Confocal microscopy

*In vivo* confocal microscopy of the cornea was performed using the Confoscan 4 (Nidek technologies) considering an area of 440 × 330 mm approximately at the central cornea. Images were analyzed by the same masked investigator using the same personal computer and with the same lighting condition to avoid variations in the magnification or the contrast of the image observed. The following parameters were evaluated: activation of keratocytes, number of sub-basal plexus nerve fibers, tortuosity of these fibers, number of bead-like formations, and endothelial cellular density.

### Keratocyte activation

Evaluated in relation to a grading scale from 0 to 4, considering the reflectivity of cellular elements with respect to background^17^.

### Study of the corneal nerves

*Number of nerves*: defined as the sum of the nerve branches present in one image. *Number of beadings*: defined as the number of beadings existent in 100 mm of nerve fiber. *Grade of nerve tortuosity*: classified in four grades considering simultaneously the frequency and the amplitude of the changes in the nerve fiber direction (from Grade 0—nerve fibers appear almost straight—to Grade 4—nerve fibers appear very tortuous—showing abrupt and frequent changes in the nerve fiber direction) according to a scale^18^.

### Study of endothelium (cellular density)

Endothelial cell count on a standard area of 400 · 400 mm.

Additional data collected were demographics, history, and clinical parameters (i.e., comorbidities).

### Statistical analysis

Descriptive statistics were produced for demographic, clinical, and laboratory characteristics of cases. Mean and standard deviation are presented for normally distributed variables, and median and inter quartile range (IQR) for non-normally distributed variables, and number and percentages for categorical variables. Groups were compared with parametric or nonparametric tests, according to data distribution, for continuous variables, and with the Pearson’s chi-2 test (the Fisher’s exact test where appropriate) for categorical variables. Since most parameters were relative to both eyes, analysis to assess changes over time were performed by means of multilevel mixed models, that is, taking into account clustering per patient. Time since study start was modelled by means of cubic splines, with knots every 12 months. Two-tailed tests were used throughout. P-value significance cut-off was 0.05. Stata computer software version 14.0 (Stata Corporation, 4905 Lakeway Drive, College Station, Texas 77845, USA) was used for statistical analysis.

## Results

Ninety-three Caucasian patients were enrolled: 44 patients in PF-group, 35 in P-group, and 14 in S-group (Table 1). Most patients (71, 76.3%) were diagnosed as open angle glaucoma. The median (IQR) age was 66 [59.9–74.5] years with no difference by group (p = 0.11); no differences were observed also by gender (p = 0.86), and visual acuity (p = 0.43).

**Table 1:** Demographic and confocal microscopy data at baseline.

Variable	Category/description	Naïve to PF-tafluprost n = 44	Naïve to Preserved prostaglandins n = 35	Switched to PF-tafluprost n = 14	p-value
Gender	females	24 (54.6)	17 (48.5)	8 (57.1)	0.86
Age (years)	Median [IQR]	66.8 [61.6–74.6]	67.1 [61.4–74.9]	59.3 [57.1–67.9]	0.11
Diagnosis	POAG	26 (59.1)	27 (77.1)	12 (85.7)	0.003
Visual acuity (decimals)	Median [IQR]	1.0 [1.0–1.0]	1.0 [0.9–1.0]	1.0 [1.0–1.0]	0.99
IOP (mmHg)	Median [IQR]	18 [16–21]	18 [15–23]	16 [15–20]	0.012

Data are N% unless otherwise specified.

IQR = InterQuartile Range.

At baseline, median [IQR] IOP values were 18 (16–21) mmHg, 18 (15–23) mmHg, and 16 (15–20) mmHg, respectively (P = 0.012).

The intraocular pressure values were kept under control during the study period, after 3 years of follow-up it was 14.5 (12–18) mmHg, 14 (13–17) mmHg and 16 (14–18) mmHg, respectively.

**Figure 1:**
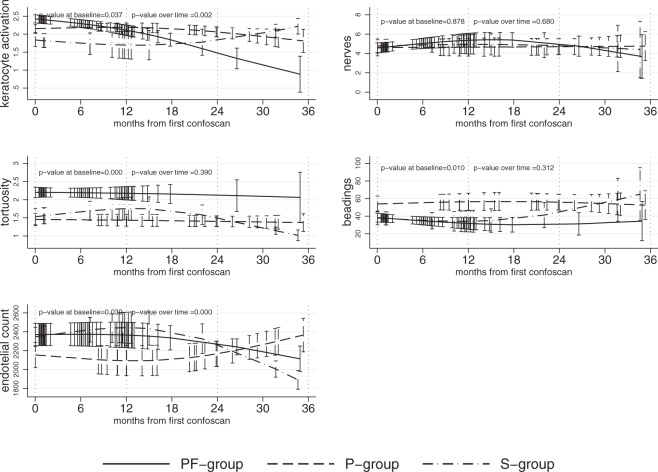
trend over time of confocal microscopy parametres in 44 patients in Preservative-Free topical therapy group, 35 in Preserved topical therapy group, and 14 in Switch group (see text). NB: multilevel mixed models taking into account clustering per patient were applied. Time since study start was modelled by means of cubic splines.

Confocal microscopy parameters differed in the three groups either at baseline (Table 2) or as trend over time (Fig. 1).

**Table 2:** confocal microscopy data at baseline.

Variable	Category/description	Naïve to PF-tafluprost n = 88 eyes	Naïve to Preserved prostaglandins n = 70 eyes	Switched to PF-tafluprost n = 28 eyes	p-value
Keratocytes activation	Median[IQR]	2 [2–3]	2 [2–3]	1.5 [1–2]	0.43
Number of sub-basal corneal nerves	Median[IQR]	4 [3–5]	3 [2–5]	5 [4–7]	0.85
Number of bead-like formations	Median[IQR]	37 [27–53]	42.5 [23–64]	34 [19–53]	0.10
Grade of tortuosity	Median[IQR]	2 [1–3]	1 [1–2]	1 [1–2]	0.0034
Endothelial count	Median[IQR]	2439 [2054–2699]	2364 [1826–2472]	2450 [2118–2559]	0.0094

Data are N% unless otherwise specified.

IQR = InterQuartile Range.

At baseline keratocyte activation was similar in the three groups (p = 0.43) but the subsequent trend over the next 36 months significantly differed (p = 0.006) with naïve patients treated with PF-tafluprost presenting a significant (p = 0.004) change from year 1 to year 3 in keratocyte activation. Sub-basal nerves tended to increase in patients switched to PF-tafluprost (p = 0.07) while they were stable in the other two groups (p = 0.11 in PF and 0.40 in P-group). Grade of tortuosity was stable over the time in the three groups. Beading-like formations were stable over the time for P- and PF-group, while in S-group a significant (p = 0.027) increase was observed.

Endothelial count values were statistically different at baseline (p = 0.007), both in PF-group and in S-group they tended to decrease (p = 0.048 and 0.001, respectively), while in P-group they increased (p = 0.0065).

## Conclusion

In this prospective study we have performed an *in vivo* confocal examination of the cornea in glaucoma patients receiving IOP-lowering treatments for 36 months. Naive patients did not show statistically significant changes from baseline regarding nerves (number, grade of tortuosity, beading like formations), but, in the PF-tafluprost group, we observed a reduction in keratocyte activation. The switch to PF-tafluprost improved the ocular surface status as measured by nerve wellness.

Medical treatment for glaucoma or ocular hypertension (OH) usually requires as initial treatment topical medication for a long period of time, in order to avoid the onset of further irreversible optic nerve damage and visual field loss. The consequences of this chronic therapy are potential adverse events and side effects, some of which may affect the ocular surface^5–8^.

For this reason, studying the modifications of the ocular surface becomes extremely important, and can be easily performed by the use of *in vivo* confocal microscopy. This diagnostic method is not invasive, quickly performed, safe and repeatable; moreover it allows a clinical insight at the cellular level to evaluate glaucoma therapy’s effects, and it is usually performed by cornea specialist^12,13,19^.

Most of the existing literature is based on cross-sectional studies, and prospective studies that evaluate naïve patients are very few, making it difficult to make comparisons. This could explain why our results partly confirm and in part differ from what is present in the literature. In 2006, Baratz *et al*. conducted a study to compare keratocyte density, sub-basal corneal nerves, and endothelial characteristics of patients with OH treated with medication or observation^14^. Thirty-eight participants underwent confocal examination: after 6 years of therapy, treated patients (n = 19) had fewer nerves and a lower nerve density than observed patients (n = 10); keratocyte density was similar. The authors, therefore, concluded that chronic administration of glaucoma medications causes a decrease in the number and density of corneal sub-basal nerve fiber bundles, but does not affect keratocyte density or corneal endothelial characteristics.

Our results found a different trend highlighting stability in the health of the nerves at 36 months follow-up. However, it should be noted that in the study of Baratz all the IOP-lowering molecules available in the market at the time of randomization were used (apraclonidine 0.5%, betaxolol 0.25%, brimonidine 0.15% and 0.2%, brinzolamide 1%, dorzolamide 2%, latanoprost 0.005%, pilocarpine 0.5% and 1%, timolol 0.5%, dorzolamide 2%/timolol 0.5% combination, and unoprostone 0.15%) whereas in our study only prostaglandin analogues (PF-tafluprost 0.0015%, P-travoprost 0.004% and P-bimatoprost 0.03%) were used.

In 2009, Martone retrospectively examined 84 patients to evaluate the long-term effects of preservative-free and preservative-containing anti-glaucoma eye drops on the ocular surface^13^. Stromal keratocyte activation and the number of beads were higher in all glaucoma preservative groups (P < 0.05). The number of sub-basal nerves was lower in all glaucoma groups than in the control group (P < 0.05) and tortuosity was significantly higher in glaucoma than control groups (P < 0.05). The authors concluded that glaucomatous patients with chronic treatment show ocular surface alterations due to therapies themselves but considered only preserved (P) latanoprost 0.005% and preserved (P) and preservative free (PF)-timolol 0.5%. A recent prospective paper of Saini and colleagues^20^ on 25 patients using two or more antiglaucoma medications and 25 controls found a significant decrease in nerve’s number, length and density in glaucoma eyes compared to controls: but patients were on more topical preserved treatments (latanoprost 0.005%, brimonidine and timolol 0.5%). Similar changes are presented by Rolle and colleagues^21^ in a cross sectional study on patients treated with PF-tafluprost 0.0015 or PF-timolol 0.1.

Our results are in complete agreement with literature regarding keratocyte activation and nerves status in glaucoma patients, and the literature adds further data, that is, a nerve layer health improvement in the S-group after the switch to PF-tafluprost with an increase of sub-basal nerves, and therefore beading-like formations, and a progressive reduction of nerve tortuosity at 36 months of treatment.

Considering that the role of active principles themselves is still unclear while it has been widely demonstrated a time- and dose-dependent toxicity of BAK on cornea and conjunctiva^5–8^, the improvements observed in the S-group are probably expression of a possible reversible effect of the ocular surface toxicity as previously stated by some authors^9,12^. The toxic effects of BAK affect, most of all, the epithelial cells that are strictly connected to the stroma affecting the nerve activity: BAK modulates the metabolic activity of nerves by creating an obstacle to healing process^9^. Moreover some authors have suggested that inflammatory mechanisms combining allergy with toxicity are involved in the complexity of reactions occurring at the ocular surface in patients using preserved topical glaucoma treatments^9^.

This information could explain what has been observed for the P-group: the number of sub-basal nerves remains stable but significantly lower than S-group and PF-group, the beading-like formations are stable on higher values than S and PF groups, as expression of a higher metabolic activity as an attempt to repair the corneal epithelial damage^14,20^. The grade of nerve tortuosity also shows stability, but the values are significantly different from the other 2 groups, as probable expression of the toxic BAK effect.

Regarding endothelial alterations, we have observed a tendency to reduce the number of cells over time, in accordance with what was previously described by Ranno *et al*. who reported a reduction of the cell density in patients treated for at least 2 years either with beta-blockers or prostaglandin derivatives^22^, but we also need to consider that endothelial cell count decreases by 10.92 cell/m2 per year^23^.

A strength of this study was the inclusion of naive glaucoma patients, and the evaluation of a group switched from a previous preserved therapy to a preservative free tafluprost therapy; however, some limitations need to be acknowledged before drawing any conclusions.

First, all confocal studies should be evaluated with caution given some limitations of this technique, such as the fact that it is a qualitative and subjective examination, the reproducibility of which has not been fully explored, even if our cornea specialist was masked to therapies. Another limitation is that the investigation is limited to the centre of the cornea, and different results might be obtained by inspecting the corneal periphery; on the other hand, previous studies refer to the same area^13^. Third, the limited number of patients precludes generalizability, and further prospective studies on a larger number of patients are needed.

In conclusion, the present study confirms the efficacy of preservative-free tafluprost in reducing IOP, and suggests that the drug can be safety used in naïve glaucoma patients with regard to corneal status as documented by confocal microscopy. PF-tafluprost has also been shown to be beneficial for the corneal health in patients switched from preserved prostaglandins.

In the balance between efficacy and safety, formulations with low cytotoxicity may ensure fewer side effects, with higher tolerability and better compliance.
